# Biofilm formation of *Clostridium perfringens* and its exposure to low-dose antimicrobials

**DOI:** 10.3389/fmicb.2014.00183

**Published:** 2014-04-22

**Authors:** Audrey Charlebois, Mario Jacques, Marie Archambault

**Affiliations:** ^1^Département de Pathologie et Microbiologie, Faculté de Médecine Vétérinaire, Université de MontréalSaint-Hyacinthe, QC, Canada; ^2^Centre de Recherche en Infectiologie Porcine et Avicole, Université de MontréalSaint-Hyacinthe, QC, Canada

**Keywords:** *Clostridium perfringens*, biofilm formation, antibiotic prophylaxis, anticoccidials, biofilms, anaerobes, low dose antibiotics

## Abstract

*Clostridium perfringens* is an opportunistic pathogen that can cause food poisoning in humans and various enterotoxemia in animal species. Very little is known on the biofilm of *C. perfringens* and its exposure to subminimal inhibitory concentrations of antimicrobials. This study was undertaken to address these issues. Most of the *C. perfringens* human and animal isolates tested in this study were able to form biofilm (230/277). Porcine clinical isolates formed significantly more biofilm than the porcine commensal isolates. A subgroup of clinical and commensal *C. perfringens* isolates was randomly selected for further characterization. Biofilm was found to protect *C. perfringens* bacterial cells from exposure to high concentrations of tested antimicrobials. Exposure to low doses of some of these antimicrobials tended to lead to a diminution of the biofilm formed. However, a few isolates showed an increase in biofilm formation when exposed to low doses of tylosin, bacitracin, virginiamycin, and monensin. Six isolates were randomly selected for biofilm analysis using scanning laser confocal microscopy. Of those, four produced more biofilm in presence of low doses of bacitracin whereas biofilms formed without bacitracin were thinner and less elevated. An increase in the area occupied by bacteria in the biofilm following exposure to low doses of bacitracin was also observed in the majority of isolates. Morphology examination revealed flat biofilms with the exception of one isolate that demonstrated a mushroom-like biofilm. Matrix composition analysis showed the presence of proteins, beta-1,4 linked polysaccharides and extracellular DNA, but no poly-beta-1,6-*N*-acetyl-D-glucosamine. This study brings new information on the biofilm produced by *C. perfringens* and its exposure to low doses of antimicrobials.

## INTRODUCTION

The predominant organizational state of bacteria in nature is biofilms. They have been defined as a structured community of bacterial cells enclosed in a self-produced extracellular matrix composed primarily of exopolysaccharides (Costerton et al., 1999). The process of biofilm formation involves the following stages: attachment, maturation and dispersion (Davey and O’Toole, 2000; Hall-Stoodley and Stoodley, 2009). Features of cells in biofilms include: aggregation in suspension or on solid surfaces, increased antibiotic resistance, protection from phagocytosis and immune cells, and resistance to physical and environmental stresses (Davey and O’Toole, 2000; Davies, 2003; Hall-Stoodley and Stoodley, 2009). In *Pseudomonas aeruginosa,* extracellular DNA present in the matrix was shown to chelate cations and to induce the expression of antibiotic resistance (Mulcahy et al., 2008). Also, promotion of biofilm formation was observed through the ability of extracellular DNA to chelate Mg^2^^+^ (Mulcahy and Lewenza, 2011). Recently, current knowledge of bacterial biofilms in animal pathogens was reviewed (Jacques et al., 2010) and surprisingly, very little is known about the biofilm formed by *Clostridium perfringens*.

*Clostridium perfringens* is a Gram-positive anaerobic bacterium that causes numerous human and animal diseases, primarily as a result of its ability to produce many different toxins (Markey et al., 2013). A classification based on the production of four major toxins (alpha, beta, epsilon, and iota) divides the *C. perfringens* into five toxigenic biotypes (A to E; Petit et al., 1999). *C. perfringens* has been ranked by the Center for Disease Control and Prevention as one of the most common bacterial causes of food-borne illness in the United States, causing nearly a million cases each year, and is also classified as a class-B bioterrorism agent (Scallan et al., 2011). Recently, an increasing number of reports have implicated the organism in antibiotic–associated diarrhea and sporadic diarrhea cases in humans, as well as diarrhea cases in animals (Smedley et al., 2004; Uzal et al., 2012; Banaszkiewicz et al., 2013). In chickens and swine, *C. perfringens* causes enteritis, a disease of economic importance to the worldwide animal-food producing industry in terms of both animal loss and vaccination costs (Markey et al., 2013). Isolates of animal origin constitute a risk for transmission to humans through the food chain. Recent studies have shown early signs of acquired antibiotic resistance in *C. perfringens* indicating that antibiotic resistance is now emerging (Lyras et al., 2009; Soge et al., 2009; Slavić et al., 2011; Charlebois et al., 2012). *C. perfringens* has been the subject of considerable investigations in the past several years; however, an area of substantial uncertainty relates to biofilm formation with, or without exposure to low-doses of anticoccidials and antibiotics. Anticoccidials are ionophores such as monensin, narasin, and salinomycin that are used to prevent/treat infections caused by coccidia, an obligate intracellular parasite, and in some cases for their effect against *C. perfringens*. These compounds are also used as growth promoters in some countries (Callaway et al., 2003; Lefebvre et al., 2006; Diarra et al., 2007).

Only a few studies have reported on *C. perfringens* biofilm formation (Varga et al., 2008; Donelli et al., 2012). Type IV pilus (TFP)-dependent gliding motility and the catabolite control protein (CcpA), a key regulator of the response to carbohydrate limitation, were both shown to be necessary for efficient biofilm formation (Varga et al., 2008). In the same study, the authors also observed that biofilm cells demonstrated 5- to 15-fold-increased survival rate over planktonic cells after exposure to penicillin G and an increased survival to oxygen stresses. It is suspected that biofilms of* C. perfringens* may play an important role in resistance to environmental stresses (Varga et al., 2008). In a study by Ledder et al. (2008) on coaggregation among numerically and ecologically important intestinal bacteria, and between intestinal bacteria and oral isolates, *C. perfringens* scored the highest overall for coaggregation among the gut species tested (Ledder et al., 2008). To our knowledge, no other data is available on biofilms of *C. perfringens*.

Studies demonstrating that low-doses of antibiotics induce bacterial biofilm formation have recently been reviewed by Kaplan (Kaplan, 2011). In contrast, low dose of a fluoroquinolone caused a significant decrease in adhesion and biofilm formation by *Stenotrophomonas maltophilia* (Pompilio et al., 2010). Also, lower cell densities within biofilms have been reported with sub-MIC of dicloxicillin, a β-lactam antibiotic, for *Staphylococcus epidermidis* and *Staphylococcus haemolyticus* (Cerca et al., 2005). The effect of sub-MIC of bacitracin, virginiamycin, lincomycin, tylosin, and ionophores on biofilm formation has not yet been described in *C. perfringens*. These antibiotics and ionophores are largely used in swine and poultry production for therapy, prophylaxis purposes, or as feed additives (low-dose usage) in some countries.

The aims of this study were to evaluate the biofilm formation of field isolates of *C. perfringens* of various sources, to determine biofilm tolerance to oxygen and antibiotics, and to investigate the effect of low doses of antibiotics and ionophores on biofilm formation.

## MATERIALS AND METHODS

### BACTERIAL ISOLATES

Commensal isolates of *C. perfringens* from poultry and swine were recovered from the normal intestinal microbiota of animals taken at seven (five poultry and two swine) processing plants located in the province of Québec, Canada. Isolates were identified and typed by PCR as previously described (Charlebois et al., 2012). Clinical isolates of animal origin were provided by the Clinical Laboratory of Molecular Diagnostic of Université de Montréal (St-Hyacinthe, QC, Canada) and human isolates were provided by the Infectious Disease Research Center of Université Laval (Québec, Canada). Thawed isolates were grown on Columbia agar with 5% sheep blood (Oxoid, Nepean, ON, Canada) and then incubated in anaerobic condition at 37°C.

### BIOFILM GROWTH AND QUANTIFICATION

Different temperatures, incubation times and growth media with and without glucose, as well as three different isolates (ATCC 13124, c1261A, FMV-CP12) were used to standardize the biofilm formation assay. More specifically, overnight blood agar cultures of *C. perfringens* were resuspended at a density of 0.5 MacFarland in Trypticase-peptone-glucose (TPG), fluid thioglycolate (FTG), tryptic soy broth (TSB; BD, Mississauga, ON, Canada) supplemented or not with 10 mM of filter-sterilized glucose (Sigma, Oakville, ON, Canada), or Brain Heart Infusion (BHI) supplemented or not with 10 mM of filter-sterilized glucose. 100 μL of cultures were added in 96-well polystyrene tissue culture plates (Costar^®^ #3595, Corning Incorporated, Corning, NY, USA) which were then incubated anaerobically at 30, 35, or 44°C for 1, 3, or 6 days in a sealed container. All isolates recovered in this study were tested for biofilm formation. These experiments were done in triplicate and repeated three times. Optimized biofilm growth conditions were as follows: bacterial cells were inoculated in TSB medium supplemented with 10 mM of filter-sterilized glucose and plates were incubated for 6 days at 44°C in an anaerobic environment. To quantify the biofilm formation, the crystal violet assay was used as described elsewhere (Varga et al., 2008). *C. perfringens* ATCC 13124 was used as positive control because this strain was previously shown to produce biofilm (Varga et al., 2008).

Isolates were categorized as described previously (Stepanovic et al., 2007). Briefly, isolates were divided into the following categories: no biofilm producer, weak biofilm producer, moderate biofilm producer and strong biofilm producer, based upon the previously calculated optical density (OD) values measured at 570 nm: OD ≤ ODc = no biofilm producer; ODc < OD ≤ 2× ODc = weak biofilm producer; 2× ODc < OD ≤ 4× ODc = moderate biofilm producer; 4× ODc < OD = strong biofilm producer. ODc is defined as three standard deviations (SD) above the mean OD of the negative control. Isolates were also compared in regards to a few parameters: clinical or commensal and animal sources. A subgroup of *C. perfringens* isolates (*n* = 18) was randomly selected for further characterization (**Table 1**). These were from the collection of isolates recovered in this study from seven processing plants. To those was added *C. perfringens* ATCC 13124 as a positive control.

**Table 1:** *Clostridium perfringens* selected isolates and their MIC (μg/mL) to the antibiotics and anticoccidials tested in this study.

				MIC (μg/mL)								
Species	Strains	Origin	Biofilm formation	Bacitracin	Virginiamycin	Penicillin	Tylosin	Lincomycin	Salinomycin	Narasin	Monensin	Source
Poultry	c1261_A	Commensal	Weak	512	0.12	0.03	0.5	16	0.12	0.12	0.25	This study
	c2188_B	Commensal	Moderate	512	0.12	0.03	0.5	32	0.12	0.06	2	This study
	c3336_B	Commensal	No	4	1	0.06	128	512	0.12	0.03	1	This study
	c3342_A	Commensal	Moderate	2	1	0.12	128	8	0.25	0.12	0.25	This study
	c3342_B	Commensal	High	256	1	0.0075	128	64	0.25	0.12	1	This study
	c3437_A	Commensal	Moderate	6	2	0.015	128	256	0.25	0.06	2	This study
	c3807_A	Commensal	No	256	1	0.03	64	8	0.25	0.06	2	This study
	STF2003-1256	Clinical	Weak	4	1	0.06	0.5	256	0.25	0.06	0.5	Dre Martine Boulianne
	2006-4758	Clinical	Weak	256	2	0.03	0.5	16	0.06	0.03	0.5	Dre Martine Boulianne
	SHY07-383	Clinical	High	3	0.5	0.0019	0.5	8	0.015	0.03	0.015	Dre Martine Boulianne
	CP4	Clinical	Moderate	1.5	0.5	0.06	1	8	1	0.25	2	Dr. John F. Prescott
	JGS4143	Clinical	Moderate	8	2	0.015	1	32	0.5	0.06	1	Dr. Glenn Songer
Human	CCRI-16276	Clinical	Moderate	16	0.5	0.03	2	8	0.5	0.25	2	Dr. Maurice Boissinot
	ATCC 13124	Clinical	Weak	2	0.5	0.03	1	0.5	0.06	0.12	0.015	ATCC
Swine	FMV-CP4	Clinical	Moderate	0.75	1	0.06	2	8	0.5	0.12	2	This study
	FMV-CP23	Commensal	No	1	0.5	0.12	1	16	0.25	0.06	1	This study
	FMV-CP71	Commensal	No	0.75	0.12	0.12	4	128	0.25	0.12	0.25	This study
	1285414	Clinical	Moderate	16	2	0.12	0.5	512	0.5	0.12	0.25	This study
	1304504	Clinical	Moderate	2	1	0.03	2	4	0.12	0.03	0.015	This study

### ANTIMICROBIAL SUSCEPTIBILITY TESTING

The subset of isolates was tested for MICs as previously described in the CLSI M11-A8 document (CLSI, 2012). Briefly, colonies of *C. perfringens,* from a 24 h culture grown at 37°C on blood agar plates (Oxoid) under anaerobic conditions, were resuspended into 5 mL of saline to achieve a 0.5 McFarland turbidity. This adjusted inoculum was then diluted 1:75 in supplemented Brucella broth. 50 μL of this suspension was then transferred to individual wells on microplates containing antibiotics. The microplates were sealed and incubated in an anaerobic chamber for 48 h at 37°C. Antimicrobials tested were penicillin, lincomycin, virginiamycin, tylosin, salinomycin, narasin, and monensin (all from Sigma). For bacitracin susceptibility testing, the Etest technique was used as described earlier (Charlebois et al., 2012). Isolates with MIC higher than 256 μg/mL by Etest were tested with the microdilutions broth technique to determine the exact MIC. *C. perfringens* ATCC 13124 was used as a control. Breakpoint for penicillin (2 μg/mL) is available from CLSI M11-A8. There is a previously published breakpoint for bacitracin (16 μg/mL) that is widely used in the literature (Chalmers et al., 2008; Charlebois et al., 2012). No other breakpoints are available for the antimicrobials tested in this study.

### BIOFILM TOLERANCE TO OXYGEN, ANTIBIOTICS AND ANTICOCCIDIALS

A subgroup of *C. perfringens* isolates (*n* = 18) was randomly selected from the collection of isolates recovered in this study from seven processing plants. These isolates were tested for their tolerance to oxygen and antibiotics when grown as biofilms. Biofilms were cultured in triplicates as described above and the initial ATP levels were measured with the BacTiter Glo kit (Promega, Madison, WI, USA) in accordance with the manufacturer’s instructions. After 6 days of incubation, the supernatants were removed and replaced with TSB-glucose in the biofilm cultures. The supernatants of the biofilm containing the planktonic cells were centrifuged and resuspended in fresh TSB-glucose medium. Planktonic cells were then transferred to fresh tissue culture plates. For both the biofilm cultures and planktonic cells, the antibiotic and oxygen tolerance assays were performed. Briefly, plates were incubated anaerobically at 44°C with 1.5 mg/mL of bacitracin, 20 μg/mL of penicillin, 512 μg/mL of lincomycin, 4 μg/mL of virginiamycin, 256 μg/mL of tylosin, 1 μg/mL of narasin, 2 μg/mL of salinomycin, or 4 μg/mL of monensin (all antimicrobials from Sigma) for 6 or 24 h. These concentrations of antimicrobials correspond to two times the highest MIC found among the isolates of the random group. The effects of different combinations of anticoccidials and antibiotics (monensin, narasin, or salinomycin with bacitracin, tylosin, or virginiamycin) on pre-formed biofilms were also analysed. For the oxygen tolerance assay, plates were incubated aerobically at 44°C for 6 or 24 h. After the oxygen and antimicrobial treatments, the ATP levels were measured with the BacTiter Glo kit. Percentages of survival were calculated by dividing the final ATP level by the initial ATP level. Treatment with penicillin was used as a positive control. The effect of low doses of antibiotics and anticoccidials on biofilm formation was assessed on the randomly selected *C. perfringens* isolates (*n* = 18). Briefly, biofilms were cultured as described above with the exception that antibiotics were added at a concentration of 0.1× the MICs of each isolate before incubation. All plates were incubated anaerobically at 44°C for 6 days in a sealed container. All isolates were done in triplicates. To quantify the biofilm formation, the crystal violet assay was used as described above. Control wells were incubated with medium only.

### EFFECTS OF ENZYMATIC TREATMENTS ON BIOFILM FORMATION

Biofilms of the selected *C. perfringens* isolates (*n* = 18) were grown for 6 days in TSB supplemented with 10 mM of glucose as described above. Wells were washed 2x with distilled water and then filled with 100 μL of PBS containing 20 μg/mL of dispersin B (Kane Biotech Inc., Winnipeg, MB, Canada) as described by Izano et al. (2007), or 100 μg/mL of DNase I or proteinase K (Kaplan et al., 2004; Grasteau et al., 2011). Plates were incubated at 37°C for 5 min for the dispersin B or 1 h for the DNase I and the proteinase K. For the cellulase treatment, wells were filled with 120 U/mL of cellulase and then incubated at 45°C for 72 h (Jain and Bhosle, 2008). After incubation, wells were washed once with distilled water and stained with crystal violet. *Staphylococcus aureus* ATCC 25923 was used as a positive control for all treatments.

### SCANNING LASER CONFOCAL MICROSCOPY

Biofilms of the selected *C. perfringens* isolates (*n* = 18) were grown in 96-well plates as described above with or without low doses of bacitracin. Plates were incubated for 6 days at 44°C under anaerobic conditions. After incubation, wells were washed two times with PBS to remove unattached cells. 100 μL of PBS containing the fluorescent FM 1-43 stain (Invitrogen, Burlington, ON, Canada), was added to each well to allow visualization of individual bacteria by laser confocal microscopy. In addition, 50 μg/mL of calcofluor white dye (Sigma), which is specific for beta-1,3 and beta-1,4 linkages in polysaccharides, was added to each well. This stain was selected because it was previously shown to bind to *C. perfringens* biofilm matrix (Varga et al., 2008). After incubation at room temperature for 30 and 15 min, respectively, wells were washed with 200 μL of sterile distilled water. Before readings, 100 μL of sterile distilled water was added to each well. An Olympus FV1000 IX81 laser confocal microscope was used to collect three-dimensional images of the biofilms. An argon laser set at 472 nm was used to excite the FM 1-43 dye (emission green) and a UV laser at 364 nm to excite the calcofluor white dye (emission blue). The images were processed using Fluoview software (Olympus). For the matrix composition, the biofilms were cultured as described above. The fluorophores assay was done as described previously (Wu et al., 2013a). Briefly, after the incubation, the wells were filled with 100 μL of Wheat Germ Agglutinin (WGA) – Oregon Green 488, Sypro Ruby Red or BOBO-3 (all from Invitrogen). WGA was diluted 1/100 in PBS whereas BOBO-3 was diluted 1/1500 in water. Sypro Ruby Red was used as described by the manufacturer. Plates were incubated for 30 min at room temperature in the dark then washed once with water and filled with 100 μL PBS. The plates were observed by confocal microscopy. The excitation/emission wavelengths for the fluorophores were as follow: 496/524 nm (WGA), 450/610 nm (Sypro Ruby Red), and 570/602 nm (BOBO-3). The images were processed using Fluoview software (Olympus). *Staphylococcus aureus* ATCC 25923 was used as a positive control for all fluorophores.

### STATISTICAL ANALYSIS

The statistical significance (*p* value) of differences in biofilm between the animal origin or commensal and clinical isolates were calculated with an unequal variance linear model. A Student *t*-test was used for the biofilm tolerance to oxygen and antibiotics assays, the low-dose and the enzymatic treatments. A *p* < 0.05 was considered to be significant. Statistics were done with the SAS software v.9.1. (Cary, NC, USA).

## RESULTS

### BACTERIAL ISOLATES

A total of 277 *C. perfringens* isolates of poultry (clinical, *n* = 14; commensal, *n* = 136), swine (clinical, *n* = 34; commensal, *n* = 50), human (clinical, *n* = 9), and other animal origins [clinical isolates from cows (*n* = 12), sheep (*n* = 10), goats (*n* = 3), horses (*n* = 3), deer (*n* = 1), duck (*n* = 1), alpaca (*n* = 1), cat (*n* = 1), dog (*n* = 1), and hare (*n* = 1)] were isolated for this study. A total of 273 isolates were of type A and 4 of type D. Type D isolates were found in clinical samples from bovine (*n* = 1) and ovine (*n* = 3).

### BIOFILM GROWTH AND QUANTIFICATION

**FIGURE 1:**
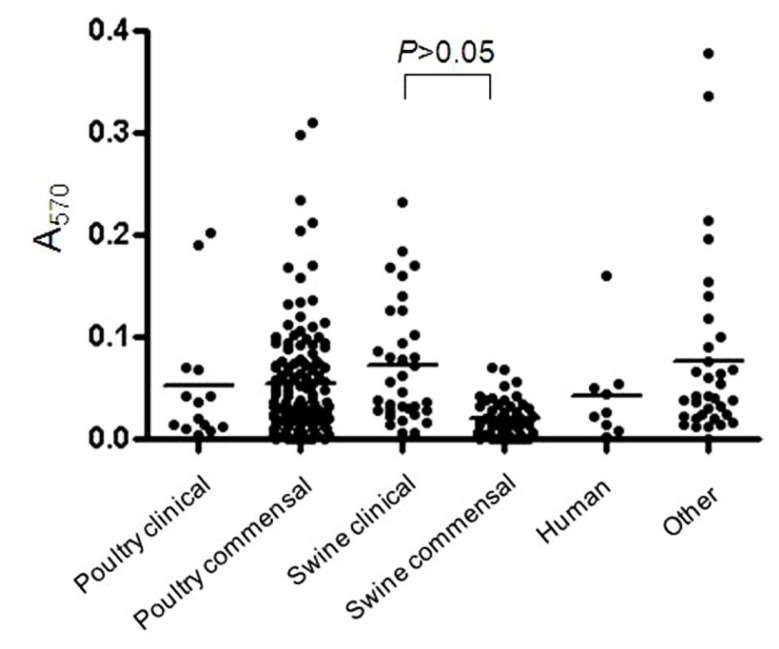
**Biofilm formation by *C. perfringens* isolates.** Biofilm formation of *C. perfringens* strains in 96-well plates, measured after 6 days as described in Section “Materials and Methods.” The *p* values were calculated using a linear model for unequal variances. Each dot represents the mean of the replicates for each strain. The bars represent the mean values of each group.

Most of the *C. perfringens* isolates tested were able to form biofilm (*n* = 230/277) in the conditions used in this study. The OD values at 570 nm (OD_570_) ranged from 0.009 to 0.489 (**Figure 1**) indicating that biofilm formation can vary among tested isolates. Of those, strong (*n* = 7), moderate (*n* = 42), and weak (*n* = 181) biofilm producers were observed. Biofilm formation was compared among clinical and commensal isolates of *C. perfringens* of animal origin to test whether there is a difference between these two groups of isolates. The mean OD value of biofilm formed by clinical isolates originating from swine was significantly higher (*p* < 0.05) than the mean OD value of their commensal counterparts (**Figure 1**). However, no difference in biofilm formation was observed between clinical and commensal isolates of poultry origin (*p* > 0.05). The low number of clinical poultry isolates was likely not sufficient to allow a statistical significance to be seen. Also, biofilm formation was compared among isolates of *C. perfringens* of different animal origins. No significant difference in biofilm formation was observed between isolates recovered from swine, poultry, human, and other animal origins (*p* > 0.05). Again, the low number of human and other animal species isolates was likely not sufficient to allow a statistical significance to be seen. A subgroup of *C. perfringens* isolates (*n* = 18) were randomly selected for further characterization (**Table 1**). These were from the collection of isolates recovered in this study from seven processing plants. To those, we included the *C. perfringens* ATCC 13124. The following categories were covered in this subgroup: commensal, clinical, swine, poultry, human, and different levels of biofilm productions. However, *C. perfringens* isolates of other animal species from this study are not represented in this subgroup.

### ANTIMICROBIAL SUSCEPTIBILITY TESTING

Isolates tested (*n* = 19) demonstrated low MICs to penicillin (0.002–0.12 μg/mL), virginiamycin (0.12–2 μg/mL), narasin (0.03–0.25 μg/mL), salinomycin (0.015–1 μg/mL), and monensin (0.015–2 μg/mL; **Table 1**). For bacitracin, 13 isolates were susceptible (0.75–16 μg/mL) and 5 were resistant (256–512 μg/mL). For lincomycin, MICs between 0.5 and 512 μg/mL were observed whereas for tylosin, MICs between 0.5 and 128 μg/mL were obtained.

### BIOFILM TOLERANCE TO OXYGEN, ANTIBIOTICS AND ANTICOCCIDIALS

**FIGURE 2:**
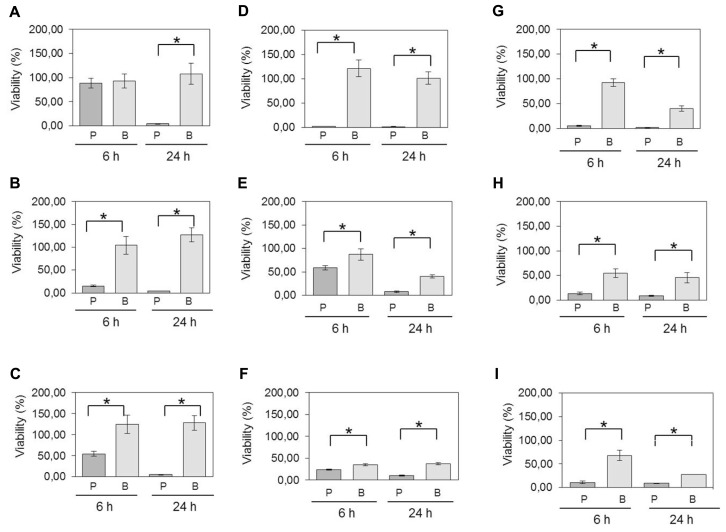
**Viability of planktonic cells compared to cells in biofilm following exposure to oxygen and antimicrobials.** *C. perfringens* 6-days-old biofilms and planktonic cultures were exposed to **(A)** atmospheric oxygen, **(B)** 1.5 mg/mL of bacitracin, **(C)** 20 μg/mL of penicillin, **(D)** 512 μg/mL of lincomycin, **(E)** 4 μg/mL of virginiamycin, **(F)** 256 μg/mL of tylosin, **(G)** 4 μg/mL of monensin, **(H)** 2 μg/mL of salinomycin, or **(I)** 1 μg/mL of narasin for the times indicated. Differences in survival of planktonic cells [P] versus biofilm [B] at each time were compared using Student’s *t*-test. *p < 0.05. The error bars represent standard deviations. Results presented are for strain *C. perfringens* ATCC 13124. For all isolates, statistically significant increases in viabilities were observed for bacteria in biofilms compared to planktonic cells.

In the atmospheric oxygen tolerance assays, the mean viability rates of planktonic cells after exposure to oxygen for 6 and 24 h were 63 and 7.4%, respectively (**Figure 2A**). However, the viability rates were higher (80.6% of viable cells after 6 h and 61% after 24 h) when *C. perfringens* cells were organized in biofilm. Data between planktonic cells and biofilm, obtained after an incubation of 24 h, were significantly different (*p*< 0.05) (**Figure 2A**). For the antibiotic and anticoccidials tolerance assays, planktonic cells had between 7.0 and 69.1% of viability after 6 h exposure and between 1.2 and 20.7% of viability after 24 h exposure to antibiotics or anticoccidials. On the other hand, cells in biofilm had between 32.7 and 65.0% of viability, and between 14.3 and 47.1% of viability for the same periods of time, corresponding to a 0.6- to 9-fold-increased survival rate after 6 h and to a 0.8- to 36-fold-increased survival rate after 24 h over planktonic cells. Viability rates for cells in biofilm were significantly higher (*p* < 0.05) than those observed for planktonic cells (**Figures 2B–I**). Antibiotics and anticoccidials were also used in combination to determine if there was a synergistic activity toward cells of *C. perfringens* ATCC 13124 within a biofilm (**Table 2**). A higher activity was observed for six combinations but these results were not significant (*p* > 0.05).

**Table 2:** Viability (%) of *C. perfringens* strain ATCC 13124 in biofilm after 24 h incubation with antibiotics and anticoccidials alone or in combinations.

	Alone	Monensin	Salinomycin	Narasin
Alone	100	65.5	79.5	61.8
Bacitracin	74.2	74.4	67.4	59.0
Virginiamycin	70.5	62.9	58.4	72.1
Tylosin	69.1	77.8	55.9	59.8

*Values underlined: values of the combination lower than the antimicrobials used alone.
*

### BACTERIAL EXPOSURE TO LOW DOSES OF ANTIBIOTICS AND ANTICOCCIDIALS

**FIGURE 3:**
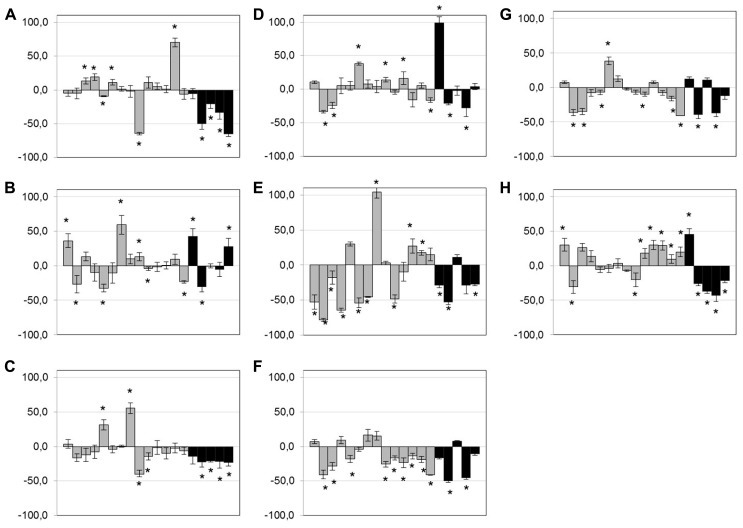
**Effect of low-dose antimicrobials on biofilm formation.** Biofilm formation of *C. perfringens* strains in 96-well plates in presence of 0.1× MIC of bacitracin **(A)**, tylosin **(B)**, penicillin **(C)**, virginiamycin **(D)**, lincomycin **(E)**, salinomycin **(F)**, narasin **(G)**, or monensin **(H)**, measured as described in Section “Materials and Methods.” Strains are in the following order: c1261_A, c2188_B, c3336_B, c3342_A, c3342_B, c3437_A, c3807_A, ATCC 13124, CCRI-16276, FMV-CP4, FMV-CP23, FMV-CP71, 1285414, 1304504, SHY07-383, STF2003-1256, 2006-4758, CP4, and JGS 4143. Results are expressed as percentages of the control not exposed to antibiotics. Conditions were compared to the control biofilm using Student’s *t*-test.*p < 0.05. The error bars represent standard deviation. Black bands represent poultry clinical isolates.

After exposure of isolates (*n* = 19) to low doses of antibiotics and anticoccidials, no clear trend was observed for bacitracin, tylosin, virginiamycin, and monensin (**Figures 3A,B,D,H**) but a few isolates showed an increase in biofilm formation when exposed to those compounds. However, exposure to low doses of penicillin, lincomycin, salinomycin, and narasin tended to lead to a diminution of the biofilm formed (**Figures 3C,E,F,G**). This phenomenon was particularly observed within the poultry clinical group of isolates. No other trends could be observed. Statistically significant results are indicated in **Figure 3** (*p* < 0.05).

### SCANNING LASER CONFOCAL MICROSCOPY

**FIGURE 4:**
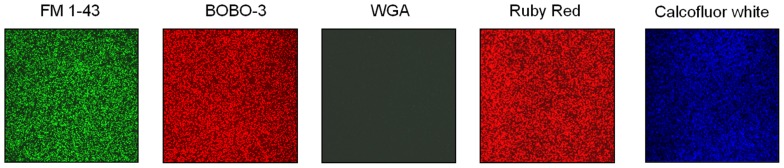
**Matrix composition of *C. perfringens* biofilm.** Representative results of the matrix composition of *C. perfringens* biofilm observed with fluorescent probes.* Staphylococcus aureus* ATCC 25923 was used as a positive control for all fluorophores. FM1-43: bacterial cells; BOBO-3: extracellular DNA, WGA: PNAG; Ruby Red: proteins; Calcofluor white: beta-1,3 and beta-1,4 linked polysaccharides.

**FIGURE 5:**
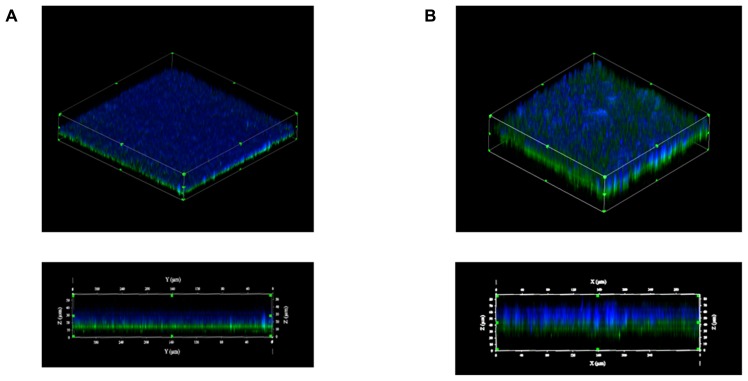
**Biofilm morphologies observed by scanning laser confocal microscopy.** Biofilms formed by the *C. perfringens* c3437_A isolate **(A)** or by the *C. perfringens* SHY07-383 isolate **(B)** after 6 days of incubation. Blue: exopolysaccharides (Calcofluor white); Green: bacteria (FM 1–43).

Using laser confocal microscopy, biofilms of tested isolates (*n* = 19) were stained with the fluorescent dyes SYPRO Ruby Red, WGA and BOBO-3 in order to visualize extracellular proteins, poly-beta-1,6-*N*-acetyl-D-glucosamine (PNAG) exopolysaccharide and DNA, respectively. Extracellular proteins and DNA were visible in biofilms of all tested isolates (**Figure 4**). However, PNAG was absent from the biofilm formed by *C. perfringens* isolates. Binding of calcofluor white to the biofilm matrix was observed. Calcofluor white binds to polysaccharides with beta-1,3 and beta-1,4 linkages. Six isolates (*C. perfringens* c1261_A, c3807_A, ATCC 13124, SHY07-383, FMV-CP23 and c3437_A) were randomly selected to further analyse their biofilms in the presence of low dose of bacitracin (0.3 × MIC). The biofilms of these isolates grown in the presence of bacitracin were approximately between 35 and 80 μm in height, whereas biofilms formed without bacitracin were thinner with elevations between 30 and 60 μm (**Table 3**). It was also found that five of them showed an increase in the area occupied by bacteria in the biofilm following exposure to low doses of bacitracin (**Table 3**). Laser confocal microscopy images showed that the biofilm formed by the *C. perfringens* isolates (*n* = 6) were mainly flat (**Figure 5A**) with the exception of one isolate demonstrating a mushroom-like biofilm (**Figure 5B**).

**Table 3:** Effect of subinhibitory concentrations of bacitracin on biofilm formation as analyzed by scanning laser confocal microscopy.

	Isolates	Total height (μm)	Matrix (μm)	Cells (μm)
Increased biofilm	c1261_A	35	18	17
	c1261_A + bacitracin	60	40	20
	c3807_A	45	35	10
	c3807_A + bacitracin	75	40	35
	c3437_A	30	22	8
	c3437_A + bacitracin	80	50	30
	SHY07-383	50	25	25
	SHY07-383 + bacitracin	55	20	35
Decreased biofilm	ATCC 13124	45	33	12
	ATCC 13124 + bacitracin	40	23	17
	FMV-CP23	60	35	25
	FMV-CP23 + bacitracin	35	20	15

### ENZYMATIC TREATMENTS ON BIOFILM FORMATION

**FIGURE 6:**
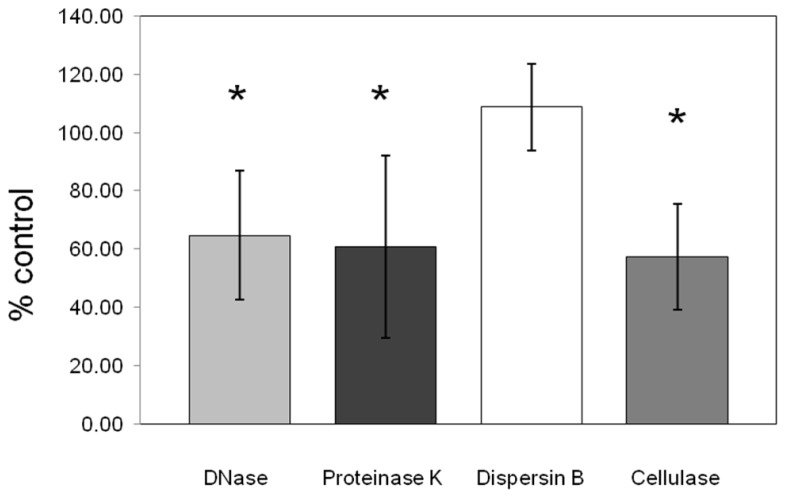
**Effect of enzymatic treatments on pre-formed *C. perfringens* biofilm.** DNase, proteinase K, cellulase, and dispersin B effects on pre-formed biofilm of *C. perfringens.* Data were measured as described in Section “Materials and Methods.” Results are the average of all strains tested and are expressed as a percentage of the control. The error bars represent standard deviation.*p < 0.05.

Dispersion of the biofilm matrix of *C. perfringens* isolates (*n* = 19) was not observed with dispersin B enzymatic treatment confirming that PNAG is absent from the matrix formed by the tested *C. perfringens* isolates. However, proteinase K, cellulase, and DNase I enzymatic treatments significantly dispersed the preformed biofilm (*p* < 0.05) indicating the presence of proteins, beta-1,4 linked polysaccharides and extracellular DNA in the matrix (**Figure 6**).

## DISCUSSION

Most of the tested isolates in this study which originated from various animal species were able to form biofilm at various degrees in the conditions used. Optimized biofilm growth conditions were different than those previously described (Varga et al., 2008; Donelli et al., 2012). However, results of this study are in accordance with the ones obtained by Varga et al. (2008) where all sequenced strains of *C. perfringens* (biotypes A of ATCC 13124, 13, and SM101), as well as representatives of type C, D, and E strains which cause infections in animals were shown to form biofilms with OD values between 0.07 and 0.5 (Varga et al., 2008). Donelli et al. (2012) were able to obtain a higher biofilm formation for *C. perfringens* strain CpeBs31 in their study, with a mean OD of 3.2, which classified this isolate as strongly adherent under the following conditions: BHI supplemented with 1% glucose and 48 h incubation at 37°C (Donelli et al., 2012). This strain was not tested in our study.

In the present study, clinical isolates recovered from swine formed significantly more biofilm than their commensal counterparts. In *Salmonella typhymurium*, it was found that clinical, outbreak-associated and retail product isolates produced thicker biofilms compared to isolates recovered from food (Castelijn et al., 2012). Reisner et al. (2006) did not observe an increased biofilm formation in human clinical strains of intestinal *Escherichia coli*. Skyberg et al. (2007) also found that isolates of *Escherichia coli* recovered from healthy birds produced significantly stronger biofilms than isolates from cases of avian colibacillosis. In *Listeria monocytogenes*, it was found that isolates from food formed more biofilm than clinical isolates recovered from cases of listeriosis (Barbosa et al., 2013). For *C. perfringens*, biofilm formation could play a role in the development of the disease because biofilm can help bacteria adhere to surfaces, and this facilitates colonization and infection. Moreover, the ability to grow as a biofilm favors survival of bacteria in the environment (Semenyuk et al., 2014). In this study, it was observed that clinical isolates of swine *C. perfringens* formed more biofilms suggesting these isolates might survive longer in the environment.

*C. perfringens* is known to be an aerotolerant bacterium capable of surviving in soil or water (Rood and Cole, 1991). Results obtained with the atmospheric oxygen tolerance assays showed that the biofilm could protect *C. perfringens* cells from oxygen stress. Varga et al. (2008) obtained similar results in their study. The involvement of biofilm in oxygen tolerance has already been described in *Fusobacterium nucleatum*, another anaerobic bacterium (Gursoy et al., 2010). The present study also showed that the biofilm could protect *C. perfringens* from high concentrations of antibiotics and anticoccidials. Increased survival of *C. perfringens* cells in biofilm following penicillin G exposure have been described elsewhere (Varga et al., 2008). Interestingly, virginiamycin, tylosin, and the three anticoccidials (namely monensin, narasin, and salinomycin) had good activity against cells in biofilm, decreasing the viability below 50%. Virginiamycin has already been found to be active against biofilms formed by some strains of *Lactobacillus* spp. (Rich et al., 2011) but to our knowledge, the effects of tylosin and anticoccidials on biofilm have never been described. Results could not be subdivided between bactericidal and bacteriostatic antimicrobials because bacteriostatic antimicrobials used at high concentrations likely become bactericidal (Pankey and Sabath, 2004). The reverse is also reported, bactericidal agents used at low doses likely become bacteriostatic (Pankey and Sabath, 2004). In the biofilm tolerance assay, antimicrobials were used at high concentrations indicating that tylosin and lincomycin are both likely acting as bactericidal in this experiment. In the bacterial exposure to low doses of antimicrobials assay, all antimicrobials likely became bacteriostatic due to the low doses used in this experiment. Moreover, in the present study, different combinations of antibiotics and anticoccidials were assessed for their activity against cells in biofilm. In general, the viability in the biofilm tended to be lower when exposed to a combination compared to either component used alone. Different combinations of antibiotics have already been described to be active against biofilm of *Staphylococcus epidermidis*, methicillin-resistant and -susceptible *Staphylococcus aureus* (MRSA and MSSA), *P. aeruginosa,* and *Enterococcus faecalis* (Saginur et al., 2006; Tre-Hardy et al., 2008; Holmberg et al., 2012; Wu et al., 2013b). This tolerance to antimicrobial agents observed in biofilm makes the treatment of these infections generally ineffective. For *C. perfringens*, it has been hypothesized that biofilm formation by this organism in the small intestine could contribute to antibiotic-associated diarrhea, a form of non-food-borne enteritis associated with antibiotic use, by facilitating in bacterial persistence through antibiotic treatment (Varga et al., 2008).

Because it has been described that low doses of antibiotics can either reduce or increase the biofilm production in other bacteria (Cerca et al., 2005; Hoffman et al., 2005; Majtan et al., 2008; Kaplan, 2011), the effect of low concentrations of antibiotics and anticoccidials on *C. perfringens* biofilm formation was studied by microplates assays. No clear trend was observed when exposed to low doses of antibiotics with the exception of penicillin, lincomycin, salinomycin, and narasin. In these cases, less biofilm was detected in the majority of isolates. Also, the effect of the antibiotics and anticoccidials on the biofilm varied depending on the strain tested indicating that this phenomenon is strain dependent. These variations among strains have also been observed for *Staphylococcus epidermidis* exposed to 0.5× the MIC of cefazolin, vancomycin, and dicloxacillin, and for *Streptococcus pyogenes* exposed to 0.015× to 0.5× the MIC of fluoroquinolones (Henriques et al., 2005; Balaji et al., 2013). To further analyse the effect of low doses of bacitracin on *C. perfringens* biofilms, laser confocal microscopy was used. An increase in the area occupied by bacteria was observed in biofilms exposed to low doses of bacitracin. This increased area occupied by bacteria has also been observed in *Staphylococcus epidermidis* biofilms exposed to 0.25× the MIC of erythromycin, in *P. aeruginosa* biofilms exposed to 0.25X the MIC of imipenem and in *Streptococcus intermedius* biofilms exposed to sub-MICs of ampicillin, ciprofloxacin, and tetracycline (Bagge et al., 2004; Ahmed et al., 2009; Wang et al., 2010).

To analyse the structure of *C. perfringens* biofilms, laser confocal microscopy was used and results obtained were consistent with the ones found in the study of Varga et al. (2008). In this study, most of the biofilms analysed in confocal microscopy were flat with one exception that showed a mushroom-like structure. To our knowledge, this is the first description of a mushroom-like biofilm in *C. perfringens*. For the matrix, it was found that the biofilm of *C. perfringens* contained polysaccharides, proteins, and extracellular DNA. Proteins and carbohydrates have already been described as components of *C. perfringens* biofilms (Varga et al., 2008). In addition to polysaccharides and proteins, extracellular DNA was also found to be a part of the matrix. The presence of extracellular DNA in the matrix of the biofilm has been described in other Gram positive bacteria (Barnes et al., 2012; Domenech et al., 2012; Kaplan et al., 2012) but to our knowledge, not in Clostridia. The mechanisms by which the DNA is released in biofilms are poorly understood but autolysis of cells has been hypothesized to mediate DNA release (Bayles, 2007; Ma et al., 2009). In *Enterococcus faecalis*, the release of extracellular DNA by autolysis is regulated by the action of the two proteases GelE and SprE (Thomas et al., 2008) whereas in *Staphylococcus aureus*, a finely tuned holin/antiholin system is thought to mediate cell lysis and programmed cell death (Bayles, 2007; Rice and Bayles, 2008). A previous study has revealed no extracellular DNA in biofilms of both *Bordetella bronchiseptica* strain 276 and *Escherichia coli* strain ECL 17602 using confocal laser scanning microscopy (Wu et al., 2013a), indicating that extracellular DNA is not a component of all biofilms. The presence of PNAG in the biofilm was studied because it is one of the most common and extensively studied matrix EPS (Jacques et al., 2010). In this study, PNAG was absent from *C. perfringens* biofilm matrix. This has been observed in other bacteria (Wu et al., 2013a). Binding of calcofluor white to the biofilm indicated that polysaccharides with beta-1,3 and beta-1,4 linkages, such as cellulose, are part of the matrix. To confirm this hypothesis, biofilms were treated with cellulase (Jain and Bhosle, 2008). It was observed that this enzyme could disperse the biofilm of *C. perfringens* confirming, for the first time, the presence of beta-1,4 linked polysaccharides in the matrix.

In conclusion, this study reports for the first time the presence of extracellular DNA and beta-1,4 linked polysaccharides in the matrix of *C. perfringens* biofilms. This study also demonstrated that virginiamycin, tylosin, and anticoccidials were active against *C. perfringens* cells in biofilm. Exposure to low doses of penicillin, lincomycin, salinomycin, and narasin tended to lead to a diminution of the biofilm formation. Further studies are needed to characterize the exopolysaccharides found in the matrix and to identify the genes involved in the biofilm formation in *C. perfringens*.

## Conflict of Interest Statement

The authors declare that the research was conducted in the absence of any commercial or financial relationships that could be construed as a potential conflict of interest.
